# Cardiac Myxoma as a Mimicker of Cerebral Vasculitis: A Case Report

**DOI:** 10.1155/crnm/8675344

**Published:** 2024-11-30

**Authors:** Nasser M. AbuDujain, Abdulaziz Alshoumar, Awyshah M. Alqahtani, Sohaila A. Alshimemeri

**Affiliations:** ^1^University Family Medicine Center, Department of Family and Community Medicine, King Saud University Medical City, Riyadh, Saudi Arabia; ^2^Division of Neurology, Department of Internal Medicine, College of Medicine, King Saud University, Riyadh, Saudi Arabia

**Keywords:** cardiac myxoma, cerebral vasculitis, misdiagnosis, stroke

## Abstract

Cardiac myxoma is considered the most common primary cardiac tumor and has been reported to cause different neurological complications through distinctive mechanisms, including pseudovasculitis. Herein, we present and review a case of a young male with a previous history of ischemic stroke who presented with multiple territorial ischemic insults in the presence of a presumed diagnosis of vasculitis. Once further workup was done, he was found to have a left atrial myxoma.

## 1. Introduction

Among all cardiac tumor types, cardiac myxoma is the commonest primary benign cardiac tumor which tends to mostly affect young women [1, 2]. It is defined by the World Health Organization (WHO) as a neoplasm composed of stellate to plump, cytologically bland, mesenchymal cells set in a myxoid stroma [2]. Although it can arise in any cardiac chamber, the left atrium is the most common location for cardiac myxomas [1]. Typically, the clinical presentation includes one or all the following: (a) symptoms secondary to cardiac outflow obstruction, (b) constitutional symptoms, and (c) symptoms related to systemic embolization (the commonest manifestation) [3]. However, it can also be asymptomatic and discovered incidentally [4]. Cardioembolic ischemic strokes are one of the leading causes of morbidity and mortality globally, attributing to around 25% of all ischemic strokes [5], although it can be prevented by early identification of the source and providing early prompt intervention. Left atrial myxoma is uncommon. However, it is a well-recognized source of embolism in cardioembolic ischemic events [6]. There are several proposed mechanisms of central nervous system (CNS) involvement in the setting of atrial myxoma. It has been described that it can be secondary to the dissemination of thrombi formed on the outer surface of the tumor. In addition, the dissemination of debris of particles from the myxoma itself can also occur [7]. Furthermore, a pseudovasculitic process induced by the cardiac myxoma has been hypothesized and has been described previously [8–12]. Here, we present a case of a young gentleman with multiple ischemic strokes secondary to atrial myxoma, who was initially thought to have vasculitis leading to his prior strokes.

## 2. Case

A 31-year-old male who had an ischemic stroke 9 months prior to his presentation to our center during which he was complaining of severe dizziness and imbalance associated with fever. His presentation was preceded by episodes of bluish discoloration of the tips of his fingers suggestive of Raynaud phenomenon associated with episodes of subjective fever that resolved spontaneously. Examination was unremarkable except for ataxic gait and positive Romberg's test. At the time of his initial presentation, he tested positive for COVID-19 on *s* PCR test and had a mild elevation of the erythrocyte sedimentation rate (ESR) to 54 mm/hr. His other laboratory tests were unremarkable. His magnetic resonance images (MRIs) brain showed multiple bilateral cerebral, cerebellar, and brainstem areas of abnormal intensity with low/isointense signal at T1WI and high signal at FLAIR/T2-WI with some of them showing restricted diffusion and areas of patchy postcontrast enhancement. His MRA was normal. A high-resolution computed tomography (HRCT) scan of the chest was done and was negative. The impression at the time was possible cerebral vasculitis. It is unclear whether the patient underwent an echocardiography during their initial presentation, and if so, whether the results were normal. In addition, all of the patient's other documentation and reports were obtained from the records provided by the nontertiary hospital where they initially received treatment. However, the patient was discharged on ASA and Atorvastatin with outpatient physical therapy and lost follow-up.

**Figure 1:**
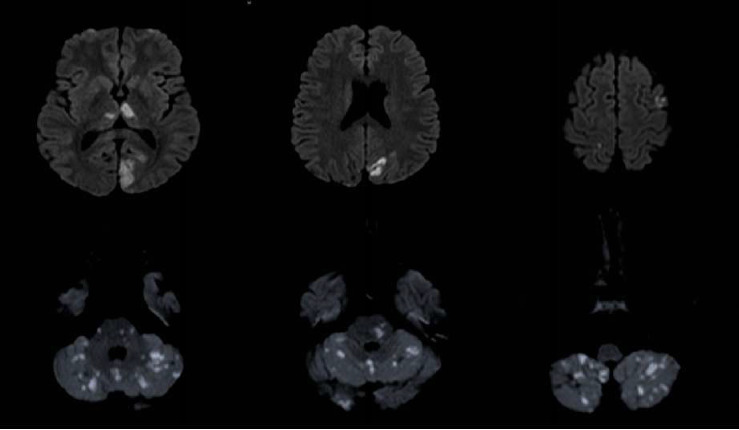
MRI diffusion-weighted images showing multiple scattered areas of diffusion restriction, in keeping with acute ischemia. The largest area is seen in the left occipital lobe. Multiple other foci are seen in other territories.

Nine months after the initial event, he was brought to our emergency department after he was found unconscious with vomitus on his shirt. An hour later, his level of consciousness improved, and his neurological examination showed moderate dysarthria, persistent hiccups, right homonymous hemianopia, restricted right eye movement in all directions, with horizontal nystagmus on left eye abduction, right upper motor neuron facial nerve palsy, and dysmetria in both his upper and lower limbs. Cardiovascular, respiratory, and rheumatological examination were unremarkable. A computed tomography (CT) brain image was immediately obtained and showed a hypodense lesion in the left caudate head and right cerebellar lobe, related to his old infarctions. CT angiography (CTA) of the brain revealed a long segment occlusion of the intracranial right vertebral artery. His MRI brain (Figure 1) showed multiple scattered areas of diffusion restriction in the posterior circulation territory, in keeping with acute ischemia involving the bilateral occipital lobes, thalami, cerebellum, and brainstem, with further scattered ischemic foci seen in the frontal, parietal lobes, and the left corona radiata with involvement of the left caudate head. His laboratory tests showed an elevated ESR level of 72 mm/hr, a high procalcitonin level (9.51 ng/mL), and a positive COVID-19 PCR test. Other serology testings including complete blood count (CBC), renal function panel, liver function tests, and C-reactive protein were normal. Antinuclear antibodies (ANAs), double stranded-DNA, SS-A antibodies SS-B antibodies, MPO and PR3, C-3 complement, cardiolipin IgG and IgM, GPI IgG, and GPI IgM were all unremarkable. His thrombophilia workup makers including factors VIII, IX, and XI and fibrinogen were normal.

**Figure 2:**
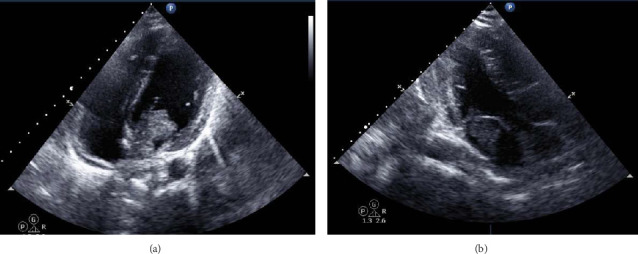
A highly mobile mass with a finger-like projection dense mass in the left atrium protruding from the mitral valve, suggestive of atrial myxoma.

An urgent echocardiogram was performed (Figure 2) and showed a highly mobile mass with a finger-like projection dense mass in the left atrium protruding from the mitral valve, suggestive of an atrial myxoma. Furthermore, a cardiac MRI was performed to further investigate the mass, which confirmed the presence of a left atrial mass with two heads, the longest reaching up to 35 mm and protruding through the mitral valve into the left ventricle. The mass is attached to the interatrial septum with a stalk with no enhancement on delayed images, confirming the diagnosis of a left atrial myxoma.

Due to the previous diagnosis of possible cerebral vasculitis at another center, a cerebral angiogram was performed and showed normal caliber arteries with no areas of stenosis, occlusion, or aneurysmal dilatation of left internal carotid, middle and anterior cerebral arteries, and cortical arteries. However, there was minor irregularity seen in the small distal branches originating from the left MCA, which is thought to be related to embolic phenomena. Based on his symptoms and the investigation findings, it was concluded that his clinical presentation is secondary to his atrial myxoma.

Ultimately, the patient was transferred to a local cardiac center where he underwent a tumor resection with no complications.

## 3. Discussion

As several studies have considered it a classical vasculitis mimicker, the diagnosis of cardiac myxoma has been a challenge, especially in the absence of cardiac symptoms. Compounding the difficulty is the similarity of symptoms and laboratory abnormalities between an atrial myxoma and connective tissue diseases, for instance, a study of 112 cases showed that 34% of the atrial myxoma had systemic signs and symptoms, including fever, weight loss, and pseudoconnective tissue disease. This resemblance makes it appear as a mimic of connective tissue disease when identified as the initial presentation. Furthermore, 37% of the same 112 cases had serological abnormalities that also mimicked those seen in connective tissue disease. These signs and symptoms may also manifest as the initial presentation in cases of atrial myxoma [3].

Throughout the years, numerous cases have been published emphasizing the diagnostic difficulty in distinguishing vasculitic phenomena associated with cardiac myxoma from a true vasculitis process [9, 13–17]. For example, a case published by Singh et al. highlighted a 1-year delay in diagnosis. This delay was attributed to the patient initially presenting as a case of multiple strokes, displaying clinical and laboratory abnormalities indicative of connective tissue disease [8]. Furthermore, in a case documented by Nishio et al. [9], a positive myeloperoxidase–antineutrophil cytoplasmic antibody (MPO–ANCA) test was reported in association with an atrial myxoma. This simultaneous presentation was referred to as pseudovasculitis. Notably, the latter case was diagnosed early because the stroke workup, which included an echocardiogram, was promptly conducted, even when there was evidence of systemic vasculitis. This emphasizes the crucial importance of consistently and thoroughly completing the stroke workup, even in the presence of a presumed etiology that is objectively apparent when investigating a patient. The same authors suggested that a true vasculitis mechanism is behind the vasculopathic phenomena in cardiac myxoma, and he explained this by the normalization of the serology level of the vasculitis markers after tumor resection which supports previous theories. However, the exact pathogenesis behind the relation of cardiac myxoma and vasculitis is still lacking. Thus, this area needs further research studies.

Table 1 presents a review and summary of comparable cases facing similar diagnostic challenges, allowing for a comparison with our case. Similar to our case, the presence of constitutional symptoms, Raynaud's phenomenon, or the painful discolored fingertips were the commonest symptoms that are suggestive of underlying systemic vasculitis [13–17]. Moreover, involvement of CNS, mainly of large vessel ischemic infarctions, has been reported in some cases at a point of the disease course, and usually later on, after the development of the systemic symptoms, similar to our case [15, 16]. The CNS manifestations vary from a transient acute neurological deficit, such as amaurosis fugax [17] to recurrent neurological deficits [9]. Interestingly, CNS manifestations can be asymptomatic, for instance, a case reported by Tan et al. showed intracranial infarctions that were discovered on an MRI scan, in a patient with atrial myxoma that did not exhibit any acute neurological symptoms [14], which raises the possibility of undiscovered CNS involvement of cardiac myxoma patients. As for peripheral nervous system (PNS) involvement, Byrd, Matthews, and Hunt presented a case of a 48-year-old male who had a painless foot drop, preceding the involvement of CNS, making associated PNS manifestations possible [16].

Laboratory findings complicate the task of distinguishing between atrial myxoma and CNS vasculitis. In our patient, the elevated erythrocyte sedimentation rate (ESR), for instance, is recognized to be elevated in cases of atrial myxomas. In addition, other serological and immunological markers such as CRP, D-DNA, IL-6, and MPO-ANCA may also be elevated, while reports indicate a potential decrease in complement levels [14–19]. Interestingly, our patient tested positive for COVID-19 during both instances of stroke presentation. While COVID-19 has been considered a cause of stroke, due to its potential to induce a prothrombotic state and endothelial injury, alongside the systemic inflammation it causes, the risk of stroke is further elevated in individuals with pre-existing vascular risk factors [20].

Although most similar cases have used corticosteroid therapy in the early course of the disease where vasculitis was the presumptive diagnosis [9–13, 15–17], we did not consider starting it since our patient did not exhibit any signs or symptoms of vasculitis with his current presentation at our center, and all his serological markers were negative in his current presentation. However, he was suspected to have vasculitis in the previous ischemic event where he had Raynaud's phenomenon (which in retrospect, could have been an embolic phenomenon to the digits from the atrial myxoma) and was evaluated by an expert rheumatologist. We are unsure whether our patient underwent an echocardiography at the facility where he was initially treated. However, he did undergo an HRCT, which can help evaluate the heart chambers and detect cardiac tumors [18]. Nonetheless, no cardiac abnormalities were detected during that assessment.

The gold standard treatment for atrial myxomas is surgical resection of the tumor which can be curative. Anticoagulation therapy can be used as a bridging therapy till surgery is carried out, and in some cases, it was considered after the surgery for secondary prevention [6]. However, due to the variability among the published cases regarding the benefit of the use of anticoagulation and the high load of the strokes and their location, we opted to avoid anticoagulation therapy in our case initially, but due to the preference of the family to delay surgical resection of the atrial myxoma, we eventually started the patient on anticoagulation therapy until his he underwent his atrial myxoma resection surgery.

## 4. Conclusion

Although cardiac myxomas are benign in nature, they can result in significant morbidity and mortality in the general population particularly in young adults. Thus, it generally represents an emergency as urgent surgical tumor resection is required and is considered curative in most cases. We report a case of a young male who developed multiple territorial ischemic strokes as a presentation of cardiac myxoma, preceded by several months by an ischemic stroke with a suspicion of systemic vasculitis. We are hoping that our case will add to the literature particularly to highlight the need to consider uncommon vasculitis mimickers as a cause of ischemic stroke, in hopes of preventing recurrence, disabling complications, and death.

## Figures and Tables

**Table 1:** Summary of our case and previously published cases of atrial myxoma misdiagnosed as vasculitis.

Study	Demographics	Clinical manifestations	CNS manifestations	Laboratory tests	Vasculitis misdiagnosis
AbuDujain et al. (Our paper)	31, male	Raynaud phenomenon, episodes of subjective fever that resolve spontaneously	Ataxia	ESR 72 mm/hr, procalcitonin (9.51 ng/mL), positive COVID-19 PCR test	Cerebral vasculitis
Fung, Edmondson, and Wald [13]	48, female	Altered LOC, central chest pain, weight loss left superficial femoral artery thrombosis, NSTEMI	None	Normocytic anemia, high CRP, high ESR, mild elevation of C3	Takayasu's arteritis
Tan et al. [14]	23, female	Frontal headache, dizziness and paroxysmal syncope, lower limb weakness, fever, arthralgia, Raynaud's phenomenon	None	Slight rise in ESR (23 mm/h) and CRP (6.96 mg/L)	Takayasu's arteritis
Sargin and Senturk [15]	32, female	Fatigue, weakness, arthralgia, myalgia, and weight loss. Ischemia of the second, third, and fourth digits of the right lower extremity	None	Anemia, high ESR and CRP, polyclonal gammopathy	Polyarteritis nodosa
Byrd, Matthews, and Hunt [16]	48, male	Constitutional symptoms, calf soreness, painful swelling of the distal fingers, and distal toes	Asymmetrical weakness and numbness, headache, blurry vision, expressive aphasia	High ESR, high Ds-DNA, low complements	Systemic vasculitis
Nishio et al. [9]	23, male	Fever, calf pain, a rash of 2 months duration, myalgia, and discoloration of the fingertips	Transient episodes of disorientation, diplopia, and vertigo, right-sided hemiparesis	Elevated CRP, elevated IgG, and IgE, elevated MPO–ANCA, elevated IL 6	Polyarteritis nodosa.
Buchanan et al. [17]	42, female	Constitutional symptoms, painful, pruritic, erythematous deep skin in the hands, stiffness, and diffuse swelling of the fingers	Amaurosis fugax	Elevated ESR and CRP	Vasculitis/subacute bacterial endocarditis

Abbreviations: CRP, C-reactive protein; ESR, erythrocyte sedimentation rate; IL, interleukin; LOC, loss of consciousness; MPO-ANCA, myeloperoxidase antineutrophil cytoplasmic antibody; MRI, magnetic resonance images; NSTEMI, non-ST-elevation myocardial infarction; PCR, polymerase chain reaction.

## Data Availability

The data that support the findings of this study are available on request from the corresponding author.

## References

[1] Blondeau P. (1990). Primary Cardiac Tumors--French Studies of 533 Cases. *The Thoracic and Cardiovascular Surgeon*.

[2] Bussani R., Castrichini M., Restivo L. (2020). Cardiac Tumors: Diagnosis, Prognosis, and Treatment. *Current Cardiology Reports*.

[3] Pinede L., Duhaut P., Loire R. (2001). Clinical Presentation of Left Atrial Cardiac Myxoma. A Series of 112 Consecutive Cases. *Medicine (Baltimore)*.

[4] Reynen K. (1995). Cardiac Myxomas. *New England Journal of Medicine*.

[5] Pillai A. A., Tadi P., Kanmanthareddy A. (2024). Cardioembolic Stroke.

[6] Yuan S. M., Humuruola G. (2015). Stroke of a Cardiac Myxoma Origin. *Revista Brasileira de Cirurgia Cardiovascular*.

[7] Lee V. H., Connolly H. M., Brown R. D. (2007). Central Nervous System Manifestations of Cardiac Myxoma. *Archives of Neurology*.

[8] Singh P. K., Sureka R. K., Sharma A. K., Bhuyan S., Gupta V. (2013). Recurrent Stroke in a Case of Left Atrial Myxoma Masquerading Vasculitis. *Journal of the Association of Physicians of India*.

[9] Nishio Y., Ito Y., Iguchi Y., Sato H. (2005). MPO-ANCA-Associated Pseudovasculitis in Cardiac Myxoma. *European Journal of Neurology*.

[10] Moreno-Ariño M., Ortiz-Santamaria V., Deudero Infante A., Ayats Delgado M., Novell Teixidó F. (2016). A Classic Mimicker of Systemic Vasculitis. *Reumatología Clínica*.

[11] Macedo M. E., Reis R., Sottomayor C. (1997). Systemic Vasculitis as the Initial Presentation of a Left Atrial Myxoma. *Revista Portuguesa de Cardiologia*.

[12] Mendoza C. E., Rosado M. F., Bernal L. (2001). The Role of Interleukin-6 in Cases of Cardiac Myxoma. Clinical Features, Immunologic Abnormalities, and a Possible Role in Recurrence. *Texas Heart Institute Journal*.

[13] Fung K., Edmondson S., Wald D. S. (2014). Atrial Myxoma Masquerading as Takayasu’s Arteritis. *Journal of the Royal Society of Medicine Open*.

[14] Tan C. Y., Qin W., Lin H., Wang Z. M., Xie Q. B., Liu Y. (2010). Atrial Myxoma With Metastasis Misdiagnosed as Takayasu Arteritis. *Rheumatology International*.

[15] Sargin G., Senturk T. (2015). Left Atrial Myxoma Mimicking Polyarteritis Nodosa. *Yonsei Medical Journal*.

[16] Byrd W. E., Matthews O. P., Hunt R. E. (1980). Left Atrial Myxoma Presenting as a Systemic Vasculitis. *Arthritis & Rheumatism*.

[17] Buchanan R. R., Cairns J. A., Kraag G., Robinson J. G. (1979). Left Atrial Myxoma Mimicking Vasculitis: Echocardiographic Diagnosis. *Canadian Medical Association Journal*.

[18] Ji X., Zhang X. (2021). Left Atrial Myxoma With Left Ventricular Myxoma Diagnosed by Ultrasound Examination: A Case Report. *Medicine (Baltimore)*.

[19] Atrial Myxoma (2017). From Diagnosis to Management.

[20] Nannoni S., de Groot R., Bell S., Markus H. S. (2020). Stroke in COVID-19: A Systematic Review and Meta-Analysis. *International Journal of Stroke*.

